# A High-Energy, Wide-Spectrum, Spatiotemporal Mode-Locked Fiber Laser

**DOI:** 10.3390/mi15050644

**Published:** 2024-05-12

**Authors:** Boyuan Ge, Yajun Lou, Silin Guo, Yue Cai, Xinhai Zhang

**Affiliations:** Department of Electrical Electronic and Engineering, Southern University of Science and Technology, Shenzhen 518055, China; geby@mail.sustech.edu.cn (B.G.); 12031116@mail.sustech.edu.cn (Y.L.); 12131034@mail.sustech.edu.cn (S.G.); caiy3@mail.sustech.edu.cn (Y.C.)

**Keywords:** spatiotemporal mode-locking, pulsed fiber lasers, multimode fibers, beam self-cleaning effect

## Abstract

In this article, we demonstrate a high-energy, wide-spectrum, spatiotemporal mode-locked (STML) fiber laser. Unlike traditional single-mode fiber lasers, STML fiber lasers theoretically enable mode-locking with various combinations of transverse modes. The laser can deliver two different STML pulse sequences with different pulse widths, spectra and beam profiles, due to the different compositions of transverse modes in the output pulses. Moreover, we achieve a wide-spectrum pulsed output with a single-pulse energy of up to 116 nJ, by weakening the spectral filtering and utilizing self-cleaning. Strong spatial and spectral filtering are usually thought to be necessary for achieving STML. Our experiment verifies the necessity of spatial filtering for achieving STML, and we show that weakening unnecessary spectral filtering provides an effective way to increase the pulse energy and spectrum width of mode-locked fiber lasers.

## 1. Introduction

Since the advent of mode-locked fiber lasers, they have been widely used in many fields due to their compactness, simplicity and stability [1]. However, the pulse energy and output power of single-mode ultrafast fiber lasers are limited by the destructive effects of nonlinearities, due to the small mode field area of the single-mode fiber [2]. To overcome these limitations, researchers proposed spatiotemporal mode-locking (STML) using multimode fibers with large mode field areas, which can simultaneously lock multiple transverse modes and longitudinal modes in ultrafast fiber lasers [3,4]. Multimode fibers that support hundreds of modes have excellent potential for expansion due to their richer spatiotemporal degrees of freedom and nonlinear effects, such as multimode solitons [5], beam self-cleaning [6] and multimode nonlinear manipulation [7]. Therefore, when placing multimode fibers into ultrafast fiber lasers, in addition to the potential advantages of increasing output energy, the spatial information and more complex nonlinear effects inherent in multimode fiber lasers will provide a huge exploration space for applications such as information optics and optical sensing.

The challenge in implementing STML lies in the simultaneous control of multiple transverse modes. Currently, it is generally believed that STML requires strong spectral and spatial filtering to control the deviation of modes resulting from the nonlinear and dispersion effects in both temporal and spectral domains [8,9], especially in the gain band of 1 µm, where the Yb-doped fiber is in the positive group-velocity-dispersion spectral range [10,11]. However, these strong filtering effects also limit the pulse energy and output power of multimode fiber lasers, making it impossible to reach the theoretical levels of STML [12]. We have demonstrated, through simulation and experiment, that spectral filtering is unnecessary for the implementation of STML, as spatial filtering alone is sufficient to achieve it. This can reduce losses and enrich the modes within the cavity.

In single-mode ML lasers, the resonant cavity only outputs a basic pulse sequence at the fundamental frequency. However, multimode ML lasers can output pulses containing three-dimensional information, i.e., pulse width, spectrum and beam profile, through different mode combinations without changing the repetition frequency. Currently, multi-pulse sequence and harmonic sequence outputs have been reported in STML [13,14,15]. However, due to the vulnerability and instability of STML, there are few experimental results for different stable single-pulse sequences. 

In this work, we present a numerical simulation and experimental realization of an STML fiber laser with a broad spectral bandwidth. The resonant cavity can achieve two different states of single-pulse mode-locking output. By weakening the spectral filtering and thus expanding the spectrum, the loss of the resonant cavity is reduced and the output energy is increased. The beam profile is optimized by the self-cleaning effect. In addition, the broad spectrum offers an advantage for subsequent amplification and compression of the pulses.

## 2. Experimental Setup of Spatiotemporal Mode-Locked Fiber Laser

**Figure 1:**
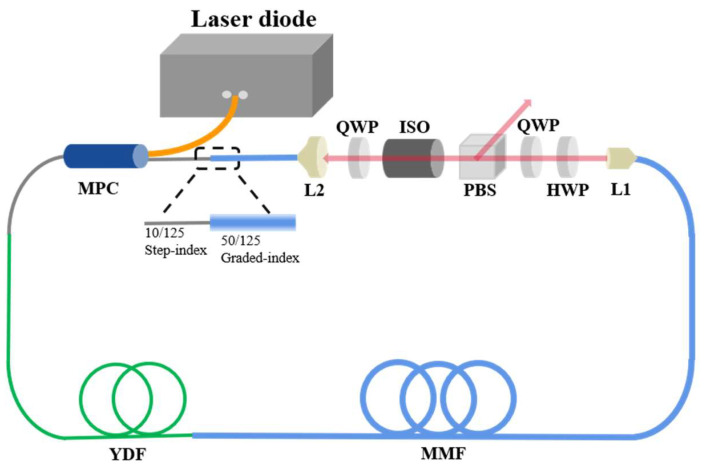
Cavity schematic of the multimode fiber laser without a spectral filter. MPC: multi-pump combiner; L1: beam collimator; L2: coupling lens; HWP: half-wave plate; QWP: quarter-wave plate; PBS: polarized beam splitter; ISO: space optical isolator; YDF: multimode gain fiber; MMF: passive graded index fiber.

The experimental setup is shown in Figure 1. The pump is provided by a 976 nm semiconductor laser, which is coupled with the ring cavity through a multimode pump combiner (MPC) with a fiber pigtail of 10/125 µm. A 2 m long Ytterbium-doped fiber (LMA-YDF-10/130-M, NA = 0.075, Nufern, Connecticut, USA) is spliced after the MPC. The tail fiber of the MPC and gain fiber both support three transverse modes at 1 µm. The passive optical fiber in the resonant cavity consists of a 5 m long graded index (GRIN) fiber (OM4-50/125, NA = 0.2, YOFC, Wuhan, China), which can support 100 transverse modes. The light emitted from the GRIN fiber is collimated by a positive lens L1 and then coupled with the cavity by another positive lens L2, after passing through the nonlinear polarization rotation (NPR) equivalent saturable absorber. It should be noted that in the enlarged region of the illustration, the spatial filtering effect introduced through the fusion of 50/125 µm and 10/125 µm fibers, similar to that at L2, is crucial for achieving STML. In contrast to the previously studied STML, the laser cavity in our experiment does not consist of additional bandpass filters, which reduces the loss of the cavity and ensures the broad bandwidth of output pulses. Additionally, our laser architecture demonstrates that spatial filtering, rather than spectral filtering, plays a decisive role in STML.

The total length of the all-multimode fiber ring cavity is 8 m. We characterize the spatiotemporal output pulses from the polarized beam splitter (PBS), by analyzing their characteristics in time and frequency domains using an optical spectrum analyzer (OSA, YOKOGAWA, Tokyo, Japan, AQ6370C), a 1 GHz oscilloscope (RIGOL, Suzhou, China DS6104), a 5 GHz photodetector (THORLABS, New Jersey, USA, DET08CFC/M), an autocorrelator (FEMTOCHROME, California, USA, FR-103XL) and an RF spectrum analyzer (RIGOL, Suzhou, China, DSA815). Additionally, a CCD (SPIRICON, Utah, USA, SP620U) is utilized to measure the beam profile of the output pulses.

## 3. Results and Discussion

**Figure 2:**
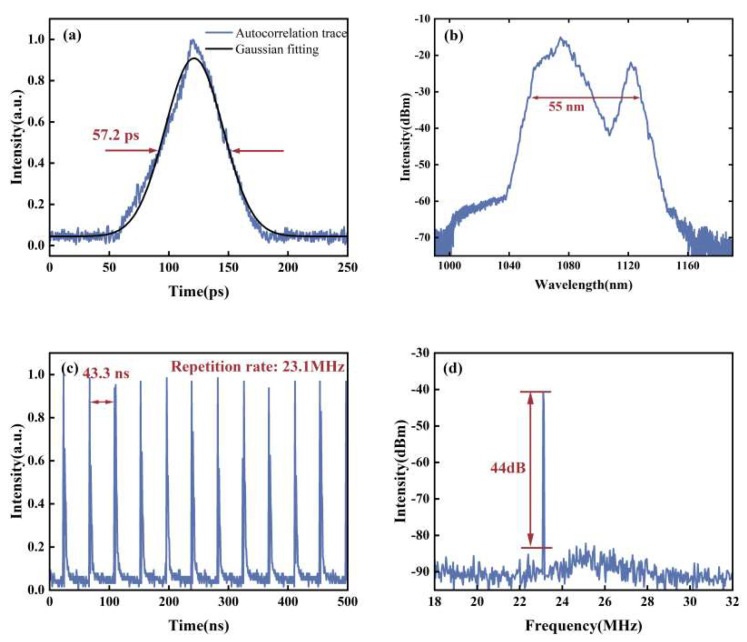
The output pulse characteristics of the first state of STML. (**a**) Autocorrelation trace; (**b**) output optical spectrum; (**c**) output pulse train; (**d**) RF spectrum.

**Figure 3:**
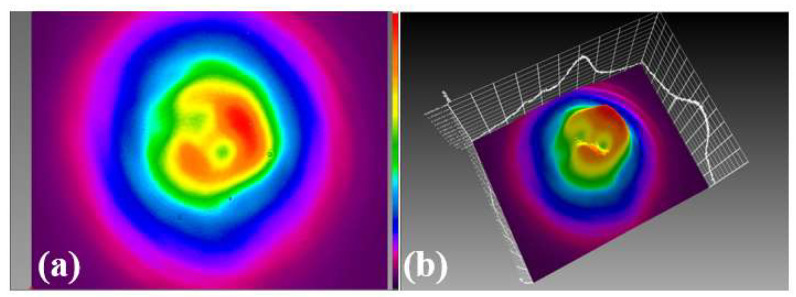
The first state of STML output pulses: (**a**) 2D and (**b**) 3D beam profiles.

By adjusting the pump power and the waveplate angle, we achieve STML with a maximum pump power of 13.95 W. Two distinct STML states are realized. The output characteristics of the first STML state are shown in Figure 2. The repetition frequency of the pulse train is 23.1 MHz, which corresponds to the length of the ring cavity. The average output power is 2.16 W, and the autocorrelation trace of a single pulse is 57.2 ps, with a single-pulse energy of 94 nJ. The signal-to-noise ratio of the RF spectrum is 44 dB. As shown in Figure 2b, the spectral width is 55 nm, with obvious double peaks, which is due to the nonlinear effects when laser pulses transport in multimode fibers. The combined effects of self-phase modulation, cross-phase modulation and mode dispersion play a major role [16,17]. The corresponding 2D and 3D beam profiles are shown in Figure 3a,b, respectively. The energy distribution of the beam profile is annular, which is significantly different from the fundamental mode.

Adjusting the angle of the waveplate will alter the polarization state of the light inside the cavity, directly affecting the losses of different transverse modes. On the other hand, the transmission function of the equivalent saturable absorber part of the single-mode fiber laser with the NPR structure is [18]

(1) $$T={Cos}^{2}\alpha_{1}{Cos}^{2}\alpha_{2}+{Sin}^{2}\alpha_{1}{Sin}^{2}\alpha_{2}+\frac{1}{2}Sin2\alpha_{1}Sin2\alpha_{2}Cos\left(∆\emptyset_{L}+∆\emptyset_{NL}\right)$$

(2) $$∆\emptyset_{NL}=-2\pi n_{2}PLCos2\alpha_{1}/\lambda A_{eff}$$

**Figure 4:**
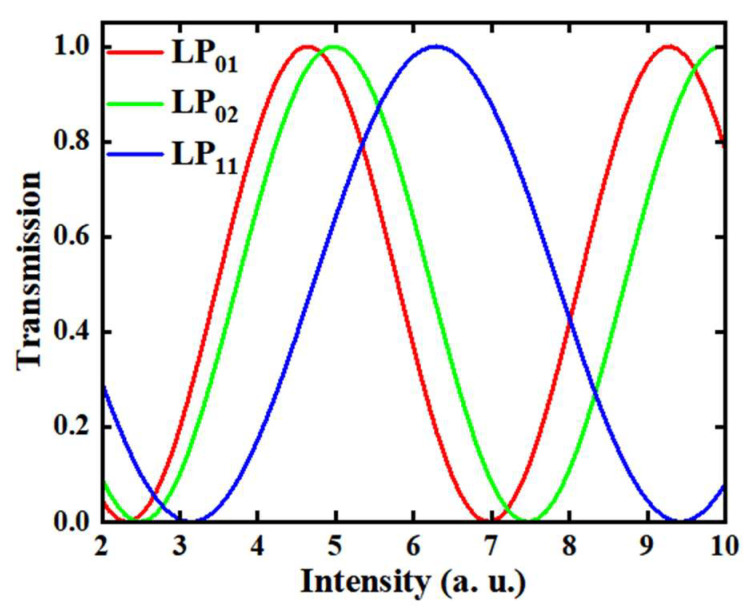
The transmittance curve distribution of the three modes (LP_01_, LP_02_ and LP_11_), obtained by normalized relative calculation based on the effective area of the LP_11_ mode.

In the nonlinear phase shift part, the effective area A*_eff_* of the mode plays a key role. Different from the single-mode fiber, the energy distribution in the multimode fiber is different due to the difference in the mode beam profile, so its effective area is also a different transmission. We calculate the effective area of three transverse modes LP_01_, LP_02_ and LP_11_ in the 50/125 µm multimode fiber, which are 967.71 µm^2^, 1037.79 µm^2^ and 1310.41 µm^2^, respectively. The obvious effective area difference in transverse modes will also bring the transmittance of SA to different modes. The relative transmittance function distribution is shown in Figure 4.

**Figure 5:**
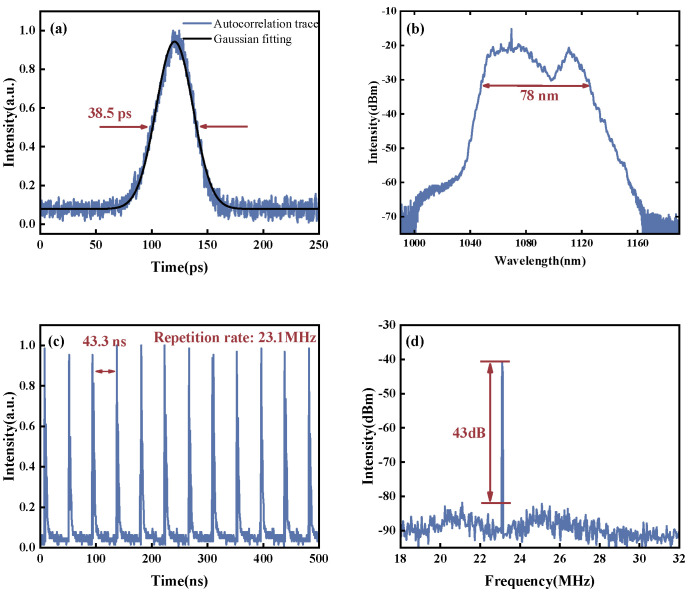
The output characteristics of the second state of STML. (**a**) Autocorrelation trace; (**b**) output optical spectrum; (**c**) output pulse train; (**d**) RF spectrum.

Meanwhile, under the effect of spatial filtering, different transverse modes will experience different filtering effects, resulting in different spatiotemporal pulse outputs with different compositions of transverse modes. This contrasts with the situations of single-mode mode-locked lasers. Principally, multimode lasers can deliver pulses with different pulse widths, spectra and beam profiles, because of different combinations of transverse modes in the laser output. By adjusting the waveplate without changing the spatial coupling conditions, we obtain the second state of STML. The tuning process is shown in Supplementary Video S1. As shown in Figure 5, the second STML output still consists of a single-pulse train. With a repetition rate of 23.1 MHz, no occurrence of multi-pulse or pulse-splitting phenomena is observed. The average power increases to 2.66 W, with the autocorrelation trajectory of 38.5 ps and the single-pulse energy of 116 nJ. Due to the increase in peak power, now, the spectral width of STML is broader than the previous one, due to stronger nonlinear effects, with a spectral width of 78 nm, as shown in Figure 5b.

**Figure 6:**
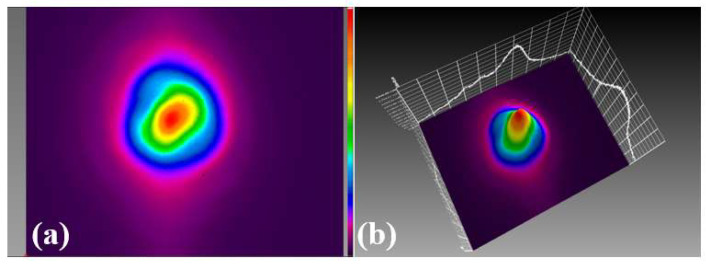
The second state of STML output pulses: (**a**) 2D and (**b**) 3D beam profiles.

Figure 6a,b show the 2D and 3D beam profiles of the STML, respectively. Compared with Figure 3, the beam energy is concentrated towards the center and the beam spot is developed towards the LP_01_ mode. The peak power of this STML state is 3 kW, which has reached the threshold of beam self-cleaning in the GRIN fiber, resulting in a beam self-cleaning effect [6,19]. We believe that the realization of two different single-pulse outputs in this laser is related to the wide spectral range allowed by the resonant cavity. The gain range of the ytterbium-doped fiber used in this experiment is 1060 nm–1115 nm. By reducing the spectral filtering, a wider gain spectrum can be obtained in the resonant cavity, so that two kinds of pulse outputs with completely different spectra, spots and pulse widths can be realized.

**Figure 7:**
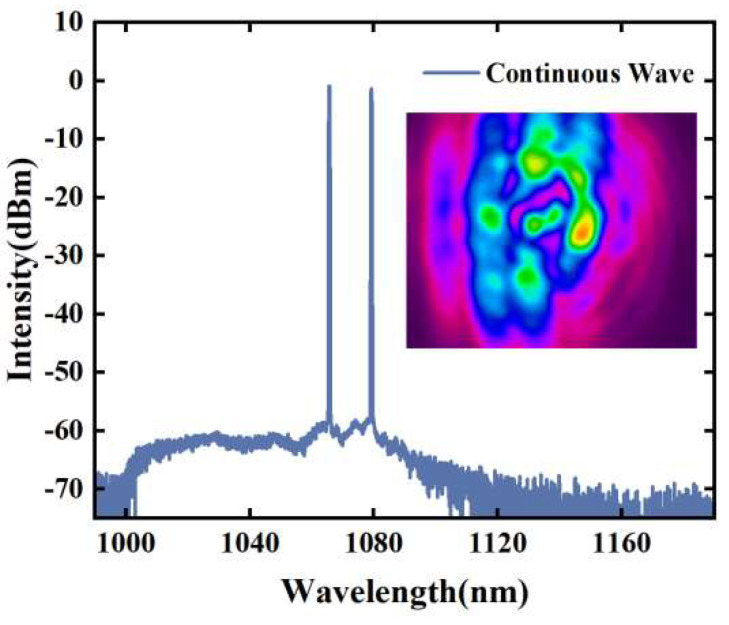
Continuous wave spectrum before STML, where the inset is the corresponding beam profile.

For comparison with the ML state, the spectra and beam profile of the continuous light before ML are presented in Figure 7. The transitions between the mode-locked and unlocked states are demonstrated in Supplementary Video S2. From the inset in Figure 7, it can be observed that the beam profile of the continuous wave exhibits strong discrete spots. Once STML is achieved, interference and coupling between transverse modes occur, and the beam profile shows a smooth distribution of energy. This energy distribution of the beam profile helps to distinguish the mode-locked state and unlocked state in multimode fiber lasers.

**Figure 8:**
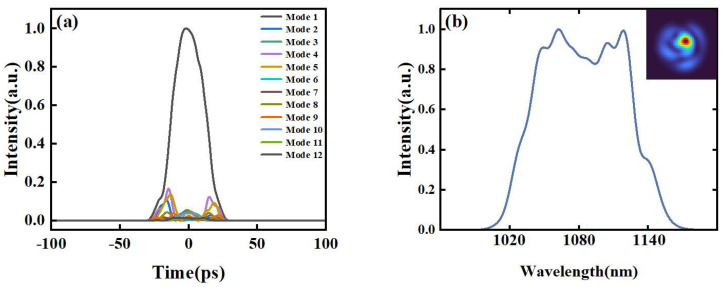
Intracavity spatiotemporal pulse propagation dynamics in simulations. (**a**) Different mode autocorrelation traces of simulated output pulses; (**b**) simulated optical spectrum of the output pulse, and the inset is the simulated output beam profile.

We conduct numerical simulations on this wide-spectrum STML fiber laser, using the generalized nonlinear Schrödinger equation [5,20]. The fiber length in the simulation model is consistent with the actual length. A 2 m long few-mode gain fiber and a 5 m long multimode graded-index fiber are used. The gain of the YDF fiber is 27 dB and the gain bandwidth is 40 nm. The optical fiber part is calculated by the nonlinear Schrödinger equation. After that, a bandpass filter with a bandwidth of 20 nm and a Gaussian-shaped spatial filter with a diameter of 30 um are set up. The above parameters are used to establish the transfer function and filter the pulse equation. The first 12 modes, ranging from low- to high-order modes, are selected in the simulation. This simplified model obtains the basic characteristics of pulse propagation within the laser. Due to the use of a GRIN fiber as the passive fiber in the laser, most of the energy is concentrated in the LP_01_ mode under the driving of the Kerr effect [6,21]. As shown in Figure 8, the spectrum of the STML pulses exhibits an evolution of broadening towards both ends, and the spectrum contains peak features.

## 4. Conclusions

In summary, we have successfully demonstrated a high-energy, broad-spectrum STML fiber laser by overcoming the limitation of spectral filtering. Two different states of single-pulse sequences of STML outputs are achieved. The highest pulse energy reaches 116 nJ with a clean beam profile and a wide range of spectra, which provides a good foundation for subsequent amplification and compression processes. This work not only simplifies the structure of multimode fiber lasers but also promotes the development and application of multimode-locked fiber lasers.

## Data Availability

The data that support the findings of this study are available from the corresponding author upon reasonable request.

## Supplementary Materials

The following supporting information can be downloaded at: https://www.mdpi.com/article/10.3390/mi15050644/s1, Video S1: Supplementary video S1, Video S2: Supplementary video S2.
